# Overexpressed lncRNA LINC00893 Suppresses Progression of Colon Cancer by Binding with miR-146b-3p to Upregulate PRSS8

**DOI:** 10.1155/2022/8002318

**Published:** 2022-05-05

**Authors:** Jing Zhu, Chao Jiang, Hongxia Hui, Yuan Sun, Mingyue Tao, Yangqing Liu, Xiaoping Qian

**Affiliations:** ^1^Comprehensive Cancer Center, Nanjing Drum Tower Hospital Clinical College of Nanjing Medical University, Nanjing, Jiangsu 210008, China; ^2^Department of Medical Oncology, The Affiliated Huaian No. 1 People's Hospital of Nanjing Medical University, Huai'an, Jiangsu, China; ^3^Comprehensive Cancer Center, Nanjing Drum Tower Hospital, Medical School of Nanjing University, Clinical Cancer Institute of Nanjing University, Nanjing Jiangsu 210008, China

## Abstract

**Background:**

Long noncoding RNAs (lncRNAs) play a significant role in the progression and metastasis of various cancers. LINC00893 has been reported to exert antitumor effect on various cancers such as gastric cancer and thyroid cancer. Bioinformatics analysis also predicted that LINC00893 was downregulated in colon cancer. However, the clinical significance and regulating mechanism of LINC00893 in colon cancer remain unknown.

**Methods:**

Expression of LINC00893, miR-146b-3p, and PRSS8 was detected in colon cancer tissues and adjacent nontumor tissues by RT-qPCR, and clinical significance was analyzed by receiver operating characteristic curve. The regulatory mechanism of LINC00893, miR-146b-3p, and PRSS8 was investigated by dual luciferase reporter and RNA pull-down assays. Proliferation, migration, invasion, and apoptosis were measured in HCT116 and SW620 cells by MTT, EdU staining, wound healing, Transwell, TUNEL, and flow-cytometry assays. Moreover, the effect of LINC00893 on colon cancer progression was further evaluated in tumor-bearing mice.

**Results:**

LINC00893 and PRSS8 were significantly downregulated, while miR-146b-3p was upregulated in colon cancer tissues compared to control group. LINC00893, miR-146b-3p, and PRSS8 had significant diagnostic value with area under curve of 0.9383, 0.7300, and 0.9644, respectively. Overexpressed LINC00893 or silenced miR-146b-3p suppressed the proliferation, migration, and invasion while promoting apoptosis in colon cancer cells (HCT116, SW620). Moreover, miR-146b-3p overexpression reversed the inhibitory effect of LINC00893, while PRSS8 knockdown rescued the suppressive effect of miR-146b-3p inhibitor on malignant cell behaviors in colon cancer. Furthermore, the tumor growth in mice was significantly reduced by LINC00893 overexpression.

**Conclusion:**

LINC00893 overexpression suppressed the progression of colon cancer by binding with miR-146b-3p to upregulate PRSS8. LINC00893 and its downstream molecules miR-146b-3p and PRSS8 may serve as novel biomarkers and therapeutic targets of colon cancer, providing new treatment options and research approaches towards colon cancer.

## 1. Introduction

Colon cancer is a malignant tumor and one of the leading causes of cancer morbidity and deaths worldwide, posing a severe danger to human health [1]. The prevalence of colon cancer varies among countries. It is more common in developed nations than in nonindustrialized nations, and in males than females [2]. Furthermore, the incidence of colon cancer continues to increase among persons under the age of 50, and the risk rises with age. The advancements in cancer detection and therapy, however, have not brought substantial increment in survival time of colon cancer patients [3, 4]. It is imperative to explore useful biomarkers to improve the colon cancer therapy [2].

A small percentage (1.2%) of the mammalian genomes encode proteins, whereas the rest of the genome is translated to a variety of noncoding RNAs (ncRNAs) [5–8]. Small ncRNAs (20–200 nucleotides) and long ncRNAs (lncRNAs, >200 nucleotides) are the two primary types of ncRNAs [9]. lncRNAs are deemed as regulating molecules and linked to a wide variety of biological functions and disorders [10–15]. lncRNAs have been indicated to influence the expression of genes through chromatin modification, transcriptional regulation, and microRNA (miRNA) sponging. A study reported that lncRNA DNAJC3-AS1 might promote colon cancer progression by mediating the miR-214-3p/Livin axis [16]. LINC00893 overexpression suppresses the proliferation, migration, and invasion of gastric cancer cells and regulates epithelial-mesenchymal transition by binding with RBFOX2 [17]. LINC00893 inhibits proliferation and migration of papillary thyroid cancer cells by inactivation of the AKT pathway via stabilizing PTEN [18]. miR-146b-3p has previously been reported to be implicated in some malignancies and exerts oncogenic effects in the growth of tumors such as colorectal cancer and thyroid cancer [19, 20]. Moreover, miR-146b is involved in cancer metastases [21, 22]. Whereas the expression of PRSS8 is decreased in the esophagus and colorectal malignancies and is inversely related to prognosis in both tumors [23, 24]. lncRNAs play a significant role in the progression and metastasis of various types of cancer via mediating miRNAs and their targeted genes. However, the clinical significance and regulating mechanism of lncRNA LINC00893 in the development of colon cancer remain to be unknown. In this study, we aimed to explore the function and regulatory mechanism of LINC00893 in colon cancer, which may provide insight on the colon cancer therapy.

## 2. Materials and Methods

### 2.1. Patients

Thirty pairs of colon cancer and normal adjacent tissue samples were retrospectively obtained from the patients in Affiliated Huaian No. 1 People's Hospital of Nanjing Medical University. The subjects were enrolled from July 2017 to July 2020. According to the Declaration of Helsinki, the study was carried out under the approval of the ethics committee of the Nanjing Drum Tower Hospital Clinical College of Nanjing Medical University. All patients had signed the informed consents before the conduction of the study.

### 2.2. Cell Culture and Transfection

Human COLO 320, SW620, LOVO, HCT116, and LIM1863 colon cancer cell lines were used in the present study. COLO 320, SW620, and LOVO were purchased from MINGZHOUBIO (Ningbo, China). HCT116 was purchased from ATCC. LIM1863 was purchased from CellBank Australia. Cells were cultured in Rosewell Park Memorial Institute (RPMI-1640) medium (Thermo Fisher, MA, USA) with 10% fetal bovine serum (FBS) (Gibco, NY, USA) and penicillin in an incubator at 37°C with 5% CO_2_. Further, the cells were seeded into the 12-well plates. LINC00893 overexpression (LINC00893-OE) plasmid, empty vector (pcDNA3.1), miR-146b-3p mimics, miR-146b-3p inhibitors, siRNA(si)-PRSS8, si-negative control (si-NC) plasmid, and blank control were transfected into colon cancer cells by Lipofectamine 3000 (Invitrogen, CA, USA) for 48 h. These plasmids were provided by GenePharma Co., Ltd. (Shanghai, China).

### 2.3. RNA Isolation and RT-qPCR

After homogenization with guanidine isothiocyanate, total RNA was collected using phenol-chloroform solutions (EP013, TRIpure Total RNA Extraction Reagent, ELK Biotechnology, Wuhan). The concentrations of RNA were determined using a spectrophotometer (ND1000, NanoDrop Technologies, USA). The total RNA was then reverse-transcribed into cDNA using the reverse transcript kit (EQ003, ELK Biotechnology, Wuhan). The following were the thermal cycle conditions involved in the PCR reaction: predenaturation was carried out for 10 minutes at 95°C; denaturation was carried out for 15 seconds at 95°C; annealing was carried out for 15 seconds at 60°C; and elongation was carried out for 20 seconds at 72°C, for a total of 40 cycles. When the temperature reached 4°C, the processes were halted. For each specimen, these three steps were followed, and quantitative analysis of the data was performed based on the 2^-△△CT^ method [25]. The primer sequences were listed as follows: LINC00893, F: 5′-CAGATCTCCATGCAAAGTATGTC-3′, R: 5′-GTTAGAATTATCTTCAAGGAGCCTC-3′; miR-146b-3p, F: 5′-GCCCUGUGGACUCAGUUCUG-3′, R: 5′-CTCTACAGCTATATTGCCAGCCAC-3′; PRSS8, F: 5′-CTATGAAGGCGTCCATGTG-3′, R: 5′-CATAGGCTTCCTTGTGGTG-3′. GAPDH, F: 5′-TCATTTCCTGGTATGACAACGA-3′, R: 5′-GTCTTACTCCTTGGAGGCC-3′; U6,F: 5′-ATACAGAGAAAGTTAGCACGG-3′, R:5′-GGAATGCTTCAAAGAGTTGTG-3′.

### 2.4. MTT Assay

MTT assay was carried out to evaluate the viability of colon cancer cells following the manufacturer's protocols (Beyotime Institute of Biotechnology). HCT116 and SW620 cell lines were incubated in 96-well plates at the density of 5 × 10^3^ cells/well and then treated with LINC00893 OE, empty vector, miR-146b-3p mimics, miR-146b-3p inhibitors, si-PRSS8, and si-NC plasmid for 24 h. Next, 10 *μ*L of MTT reagent (Beyotime, Shanghai, China) was added into each well and further incubated for 4 h at 37°C. The optical density (OD) value was evaluated by a microplate reader (Promega, WI, USA) at 450 nm. Results from three independent experiments were normalized to the control group and expressed as the mean ± SD.

### 2.5. EdU Assay

The 10 *μ*mol/L EdU was added into a growth medium and cultured in the incubator for 24 h after cell transfection. In the following step, 1 mL of 0.5% triaxone solution was added, followed by 0.5 mL reaction solution and 0.5 mL Hoechst 33342 solution for 30 minutes of incubation, after which the photographing and counting of the stained cells were carried out using an inverted fluorescent microscope.

### 2.6. Wound Healing Assay

After the cell transfection, the colon cancer cells were treated with trypsin, plated into the 6-well plates, and incubated till reaching 80% confluence of the medium. Then, the sterile pipette (200 *μ*L) tip was used to scratch each well followed by washing with PBS solution several times to abolish cell debris. In the following 48 h, cells were incubated in medium (serum-free), and the migrated cells to the surface of the wound were considered as fabricating an *in vitro* healing process. The images of the wound healing were obtained by an inverted microscope (Olympus Corporation, magnification: ×100), and the closure rate was evaluated. Wound width was assessed by an Image J software (National Institutes of Health, MD, USA). Wound healing rate was calculated as the (width at 48 h − width at 0 h)/width at 0 h × 100%.

### 2.7. Transwell Assay

Matrigel was equally spread on the transwell chamber's bottom surface (Corning, Shanghai, China). 5 × 10^4^ cells were added to the upper chamber. 500 *μ*L of medium containing 10% FBS was put into the lower chamber. Then, cells on the upper chamber were wiped away. Afterward, the culture medium was discarded, and cells were stained with crystal violet for 5 minutes. Subsequently, after washing with PBS and drying, images were taken by a microscope (Olympus, Japan).

### 2.8. Flow-Cytometry Analysis

A Cell Cycle Kit (Beyotime, China) was used to conduct cell cycle analyses. Immediately following transfection, the cells were extracted and fixed in 70% ethanol for 2 h at 4°C. A centrifuge was used to treat these cells for 5 minutes at 1000 g. These cells were then treated with 25 *μ*L of propidium iodide liquid and 10 *μ*L of RNase A for 30 minutes at 37°C. The cell cycle was measured using the NovoCyte Flow Cytometer (ACEA Biosciences, China).

### 2.9. TUNEL Apoptotic Assay

Apoptosis of cells after indicated transfection was examined under various circumstances using a TUNEL apoptosis kit (Roche, Basel, Switzerland). In brief, cells were fixed in 4% paraformaldehyde. Afterward, the cells were cleaned with PBS and treated with a fluorescein-labeled TUNEL reaction mixture in a moistened chamber for 1 h at room temperature. A fluorescent microscope was used to count the number of apoptotic cells (×200). Subsequently, apoptotic cells were counted under an optical microscopy.

### 2.10. Dual-Luciferase Assay

The binding sequences between LINC00893 and miR-146b-3p, miR-146b-3p, and PRSS8 were obtained from bioinformatics tools, LncBase V2, and TargetScan. Then, the targeted fragment was amplified from cDNA by PCR and inserted into a pmirGLO vector. Afterward, HCT116 and SW620 cells were seeded into a 96-well plate at the density of 1 × 10^5^ cells/well and cotransfected with pmirGLO vectors and miR-146b-3p mimics using 3000 Lipofectamine (Invitrogen, Carlsbad, USA). Forty-eight hours posttransfection, relative firefly luciferase activities were standardized to luciferase activities of Renilla.

### 2.11. Pull-Down Assay

miR-146b-3p was labeled with biotin and was transfected into HCT116 and SW620 cells with bio-NC for 48 h. At the end of the process, the cells were lysed, and the complex was incubated with streptavidin magnetic beads for 4 h followed by rinsing three times with wash buffer. Following extraction with Trizol, RT-qPCR assay was used to assess the attached RNA in the complex.

### 2.12. Western Blot

The cells were kept in a 6-well plate after transfection for 48 h. Then, the cells were put into the RIPA lysis buffer (Beyotime, China). Protein with equal amounts was separated by SDS-PAGE and transferred onto PVDF membranes (Merck Millipore, MA, USA). Then, the membranes were blocked with skim milk (5%), cleansed with Tris-buffered saline, and incubated with primary antibodies overnight at 4°C. The primary antibodies included PRSS8 (1/1000, ab185236, Abcam, MA, USA) and GAPDH (1/2000, EM1028, ELK Biotechnology, Wuhan). Next, the membranes were incubated with the secondary antibody HRP-conjugated goat anti-rabbit IgG (ab6721, Abcam, USA) for 1 h. Furthermore, the membranes were analyzed by an ECL reagent (Thermo Scientific Pierce, IL, USA). The grey density of protein bands was normalized to the internal reference GAPDH.

### 2.13. Xenograft Tumor Model

The mice (male athymic BALB/c nude) were provided by SLAC laboratory animal Center (Beijing, China). Subcutaneous injections of HT116 and SW620 cells were given to each mice in order to construct mouse xenograft models. The 5 × 10^6^ cells that were stably transfected with LINC00863-OE or an empty vector were suspended in 100 *μ*L phosphate buffer and administered subcutaneously into untreated nude mice. Mice were executed four weeks later. The ethics committee of the Affiliated Huaian No. 1 People's Hospital of Nanjing Medical University has approved the study, which follows the guidelines set out by the National Institutes of Health's Guide on the Care and Use of Laboratory Animals.

### 2.14. Bioinformatics and Statistical Analysis

Bioinformatics tool GEPIA [26] was used to create a box plot of LINC00893 expression in colon cancer. Graphpad prism and SPSS softwares Version 20.0 were used in the study for statistical analysis, and all the data were presented as the mean ± standard deviation. *T*-test, one-way, and two-way ANOVA analyses were used to compare differences in two or more groups. LINC00893, miR-146b-3p, and PRSS8 clinical significances were explored using the receiver operating curve (ROC) based area under the curve (AUC) in the peripheral blood samples. *P* < 0.05 was considered to have statistical significance.

## 3. Results

### 3.1. Validation of LINC00893, miR-146b-3p, and PRSS8 in Colon Cancer

**Figure 1:**
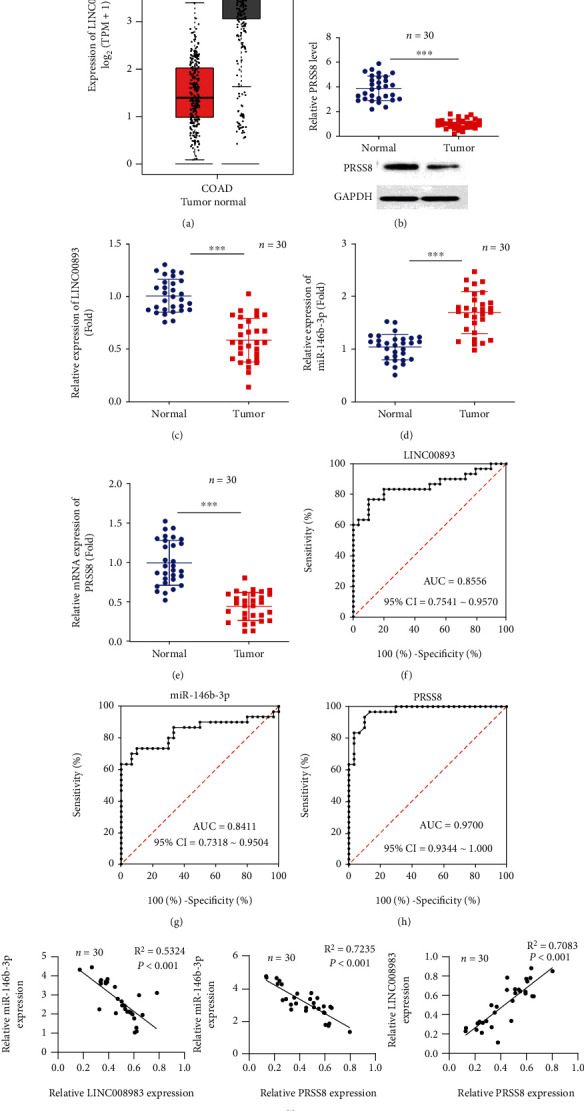
Validation of the LINC00893, miR-146b-3p, and PRSS8 in tumor tissues and adjacent normal tissues. (a) The LINC00893 expression in colon adenocarcinoma tissues based on the GEPIA tool. (b) PRSS8 relative protein expression in colon cancer tissues was measured by western blot. (c)–(e) The RNA expression levels of LINC00893, miR-146b-3p, and PRSS8 in tumor and adjacent tissue samples of colon cancer patients. (f)–(h) The clinical significance of LINC00893, miR-146b-3p, and PRSS8 was measured in colon cancer patients by ROC curves. (i) Expression correlation of LINC00893, miR-146b-3p, and PRSS8 in colon cancer tissues (*n* = 30) was analyzed. ^∗^*P* < 0.05, ^∗∗∗^*P* < 0.001.

GEPIA was used to determine LINC00893 expression in colon cancer tissues (*n* = 275) and normal adjacent tissues (*n* = 349). The box plot showed significant downregulated expression of LINC00893 in colon adenocarcinoma tissues (Figure 1(a), *P* < 0.05). Using bioinformatics analysis, the present study found that LINC00893 targeted miR-146b-3p, whereas miR-146b-3p targeted PRSS8 gene. Thus, the present study aimed to validate the molecular mechanism of LINC00893 to miR-146b-3p and miR-146b-3p to PRSS8 in colon cancer proliferation, migration, invasion, and apoptosis.

Current study has shown the expression of LINC00893, miR-146b-3p, and PRSS8 in colon cancer tissue samples (*n* = 30) and adjacent tissues (*n* = 30) by RT-qPCR. Further, PRSS8 protein expression was measured by western blot in colon cancer and adjacent tissues. The results showed that PRSS8 protein expression was downregulated in colon cancer tissues (Figure 1(b), *P* < 0.01). Furthermore, LINC00893 and PRSS8 expression were significantly downregulated while miR-146b-3p expression was upregulated significantly in colon cancer tissues (Figures 1(c)–1(e), *P* < 0.01). Clinical significance of LINC00893, miR-146b-3p, and PRSS8 was measured in colon cancer with ROC based AUC of 0.8556 (95%CI = 0.7541 ~ 0.9570), 0.8411 (95%CI = 0.7318 ~ 0.9504), and 0.9700 (95%CI = 0.9344 ~ 1.000), respectively (Figures 1(f)–1(h), *P* < 0.05). The expression correlation of LINC00893, miR-146b-3p, and PRSS8 in colon cancer tissues (*n* = 30) was analyzed, and the results showed that LINC00893 expression was negatively correlated with miR-146b-3p expression and positively correlated with PRSS8 expression. miR-146b-3p expression was found to be negatively correlated with PRSS8 expression in colon cancer tissues (Figure 1(i), *P* < 0.001). Meanwhile, the demographical parameters of colon cancer patients and their correlation with LINC00893 expression were represented in Table 1. The *χ*^2^ test analysis revealed that higher LINC00893 expression was correlated with advanced tumor stage in colon cancer (*P* = 0.0001).

### 3.2. LINC00893 Expression and Its Role in Progression, Migration, Invasion, and Apoptosis of Colon Cancer Cells

**Figure 2:**
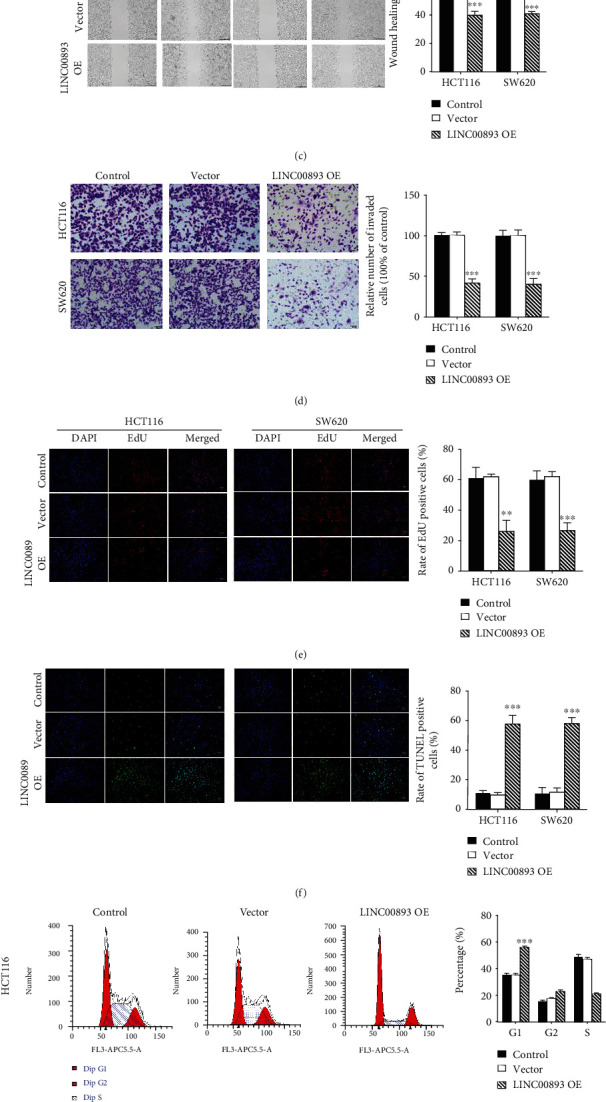
The inhibitory effect of LINC00893 overexpression on colon cancer cells. (a) LINC00893 expression in five different colon cancer cell lines and control cells was analyzed by PCR. (b) MTT assay was performed to evaluate cell viability in HCT116 and SW620 cells under the condition of LINC00893 overexpression. (c) A wound-healing assay was used to evaluate migration capacity in colon cancer cells. (d) Transwell assay was used to reveal cell invasion of HCT116 and SW620 cells in LINC00893 OE and other groups. (e) EdU assay was performed to evaluate cell proliferation of HCT116 and SW620 cells in indicated groups. (f) The apoptosis rate of colon cancer cells after indicated treatment was measured by the TUNEL assay. (g, h) Cell cycle arrest of (g) HCT116 and (h) SW620 cells after transfection with LINC00893 overexpressing vector was assessed by flow cytometry analysis. ^∗^*P* < 0.05, ^∗∗^*P* < 0.01, ^∗∗∗^*P* < 0.001.

We evaluated LINC00893 expression by RT-qPCR in colon cancer cell lines COLO 320, SW620, LOVO, HCT116, and LIM1863 cell lines and control cell line. The result demonstrated significant downregulated expression of LINC00893 in colon cancer cells (Figure 2(a), *P* < 0.05). Further, two cell lines HCT116 and SW620 were selected for subsequent *in vitro* assays. Herein, the transfections of LINC00893 OE, empty pcDNA3.1 vector were performed in HCT116 and SW620 cells to evaluate the biological regulatory functions of LINC00893. The MTT assay demonstrated that the cell viability was markedly decreased after LINC00893 OE transfection (Figure 2(b), *P* < 0.05), which suggested that LINC00893 overexpression had a suppressive effect on the proliferation of HCT116 and SW620 cells.

The key indicators for tumor metastasis are migration and invasion of cells. The present study used wound healing and Transwell assays to detect the migrative and invasive capacities of colon cancer cells. Wound healing assay showed that the LINC00893 OE significantly inhibited the migration of HCT116 and SW620 cells compared to empty vector and control groups (Figure 2(c), *P* < 0.05). Subsequently, Transwell assay was performed to determine the invasive ability of HCT116 and SW620, which showed that LINC00893 OE significantly suppressed the invasion of HCT116 and SW620 cells compared to control groups (Figure 2(d), *P* < 0.05). Meanwhile, an EdU assay showed similar suppression of proliferation in colon cancer cells by LINC00893 OE (Figure 2(e), *P* < 0.05). The TUNEL assay demonstrated that the apoptosis rate of HCT116 and SW620 cells was significantly increased in LINC00893 OE group compared to vector and control groups (Figure 2(f), *P* < 0.05). The cell-cycle arrests were demonstrated by flow-cytometry analysis, which showed that transfection of LINC00893 OE significantly increased the number of cells in G1 phase (Figures 2(g) and 2(h), *P* < 0.05). Taken together, LINC00893 may act as a tumor suppressor in colon cancer.

### 3.3. LINC00893 Targeted miR-146b-3p in Colon Cancer Cells

**Figure 3:**
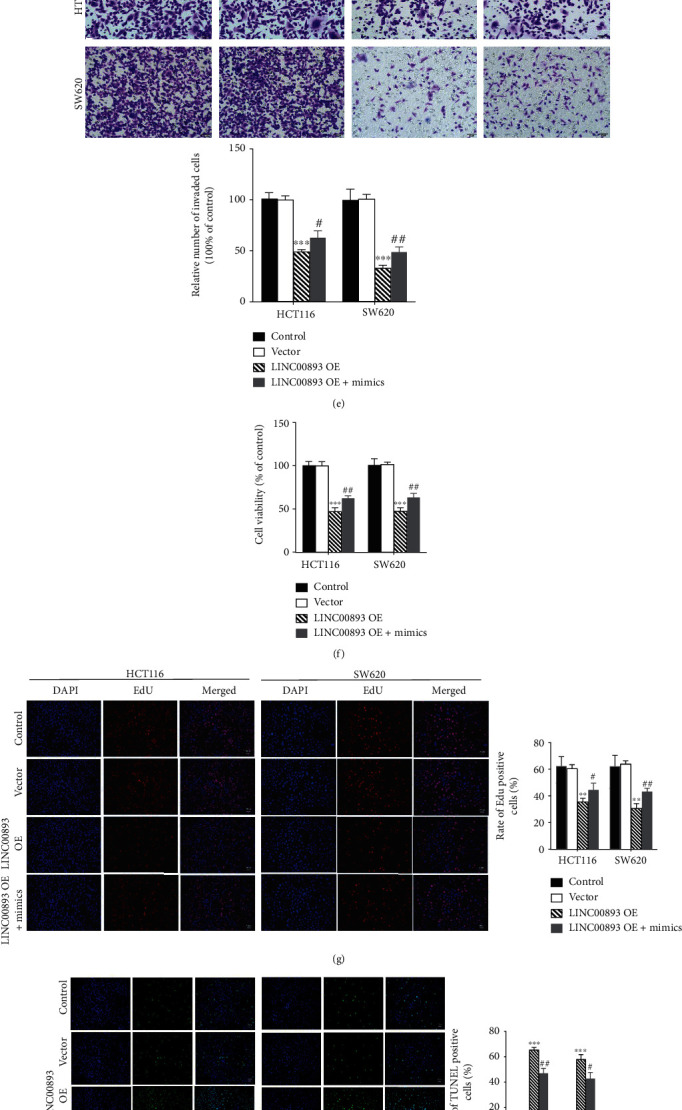
LINC00893 binds with miR-146b-3p in colon cancer cells. (a) The targeted sequence of LINC00893 to miR-146b-3p by LncBase online tool. (b) The RNA pull-down assay was carried out to evaluate the binding between LINC00893 and miR-146b-3p in HCT116 and SW620 cells. (c) Overexpression efficiency of miR-146b-3p in HCT116 and SW620 cells was assessed by RT-qPCR. (d) The dual-luciferase assays were performed to investigate the binding between LINC00893 and miR-146b-3p. (e) Transwell assay was used to detect the invasion of HCT116 and SW620 cells transfected with LINC00893 OE, LINC00893 OE + miR − 146b − 3p mimics, and empty vector. (f) MTT assay was used to detect the cell viability in four groups. (g) EdU assay showed the cell proliferation in transfected cells. (h) TUNEL assay was performed to evaluate the apoptotic ability of transfected cells in four groups. (i, j) Cell cycle arrest of (i) HCT116 and (j) SW620 cells after transfection with LINC00893 overexpressing vector and miR-146b-3p mimics was assessed by flow cytometry analysis. ^∗∗^*P* < 0.01, ^∗∗∗^*P* < 0.001, ^#^*P* < 0.05, ^##^*P* < 0.01.

The bioinformatics tool LncBase v2 [27] showed the predictive binding sequences between LINC00893 and miR-146b-3p in Figure 3(a). Furthermore, the result of RNA pull-down assays showed that LINC00893 enrichment was significantly higher in biotin-miR-146b-3p complex compared with the bio-NC groups (Figure 3(b), *P* < 0.05). Subsequently, miR-146b-3p overexpression efficiency by miR-146b-3p mimics was verified in colon cancer cells (Figure 3(c), *P* < 0.05). The luciferase reporter assay showed that the luciferase activity of WT-LINC00893 was inhibited by miR-146b-3p overexpression in colon cancer cells compared with the NC group, while that of the MUT-LINC00893 showed no significant change (Figure 3(d), *P* < 0.05).

Transwell assay was used to determine the effect of LINC00893 OE + miR − 146b − 3p mimics on invasion of HCT116 and SW620 cells. The result demonstrated that miR-146b-3p overexpression reversed the inhibitory effect of LINC00893 upregulation on the invasion of HCT116 and SW620 cells (Figure 2(e), *P* < 0.05). The MTT assay also showed that the cell viability of colon cancer cells transfected with LINC00893 OE + miR − 146b − 3p mimics was increased compared to the LINC00893 OE groups (Figure 3(f), *P* < 0.05), Meanwhile, EdU assay revealed that miR-146b-3p overexpression rescued the inhibition of LINC00893 upregulation on proliferation of colon cancer cells (Figure 3(g), *P* < 0.05). While LINC00893 OE + miR − 146b − 3p mimics showed significantly decreased apoptosis in colon cancer cells, compared to LINC00893 OE (Figure 3(h), *P* < 0.05). Moreover, the cell cycle arrests in G1 phases were significantly decreased in LINC00893 OE + miR − 146b − 3p mimics groups of colon cancer cells (Figures 3(i) and 3(j), *P* < 0.05).

### 3.4. miR-146b-3p Targeted PRSS8 and Their Roles in Colon Cancer Cells

**Figure 4:**
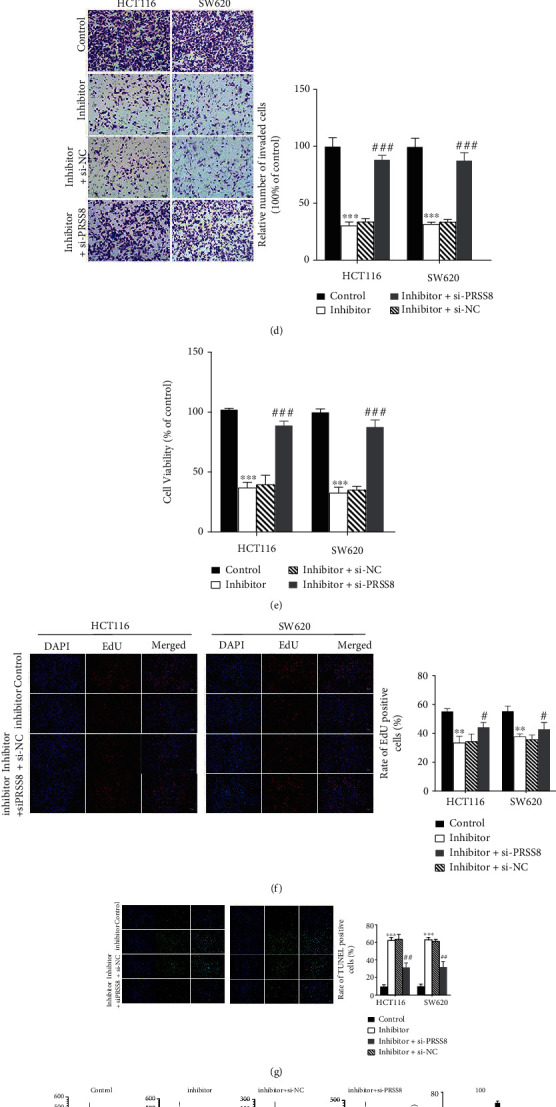
PRSS8 is a target of miR-146b-3p and their functions in colon cancer cells. (a) The binding site between miR-146b-3p and PRSS8 was predicted by Targetscan. (b) Western blot and RT-qPCR analyses were used to analyze the protein and mRNA expression of PRSS8 in si-PRSS8 (1#, 2#, 3#), si-NC, and control groups in HCT116 and SW620 cells. (c) Luciferase reporter assay was performed to investigate the binding between miR-146b-3p and PRSS8 in HCT116 colon cancer cells. (d) Transwell assay showed invasion of HCT116 and SW620 cells after transfections with miR-146b-4p inhibitors, miR − 146b − 4p inhibitors + si − PRSS8, and miR − 146b − 4p inhibitors + si − NC. (e) MTT assay represented the cell viability of four groups in transfected cells. (f) EdU assay demonstrated the cell proliferation in transfected cells. (g) TUNEL assay was performed to evaluate the apoptotic ability of four groups in transfected cells. (h, i) Cell cycle arrest of (h) HCT116 and (i) SW620 cells after transfection with si-PRSS8 and miR-146b-3p inhibitor was assessed by flow cytometry analysis. (j) PRSS8 expression in colon cancer cells transfected with LINC00893 OE. (k) PRSS8 expression in colon cancer cells transfected with miR-146b-3p mimics. ^∗^*P* < 0.05, ^∗∗^*P* < 0.01, ^∗∗∗^*P* < 0.001, ^#^*P* < 0.05, ^##^*P* < 0.01, ^###^*P* < 0.001.

The predictive targeted gene of miR-146b-3p was analyzed by Targetscan [28], and the binding site between miR-146b-3p and PRSS8 was shown in Figure 4(a). To investigate the regulatory mechanism of miR-146b-3p on PRSS8, a series of assays were performed. HCT116 and SW620 cells were transfected with si-PRSS8 (1#, 2#, #3) and si-NC, and the knockdown efficiency of PRSS8 was verified using western blot and RT-qPCR (Figure 4(b), *P* < 0.05). Moreover, the interaction between miR-146b-3p and PRSS8 was investigated by luciferase reporter assay. The result showed that the luciferase reporter activity of PRSS8-WT was significantly reduced after miR-146b-3p overexpression, while that of the PRSS8-MUT showed no significant change (Figure 4(c), *P* < 0.05).

Furthermore, miR-146b-3p knockdown significantly inhibited the migration and invasion in HCT116 and SW620 cells, which was revealed to be rescued by PRSS8 silencing (Figure 4(d), *P* < 0.05). Cell viability showed an increase in cells transfected with miR-146b-3p inhibitors + si-PRSS8, while viability of cells transfected with miR-146b-3p inhibitors was significantly decreased in the MTT assay (Figure 4(e), *P* < 0.05). Further, silenced PRSS8 reversed the inhibition induced by miR-146b-3p knockdown on cell proliferation in colon cancer cells, as revealed by EdU assay (Figure 4(f), *P* < 0.05). Meanwhile, silenced PRSS8 significantly rescued the promotive effect of miR-146b-3p inhibitors on the apoptosis of colon cancer cells (Figure 4(g), *P* < 0.05). However, the cell cycle arrests in G1 phases were increased miR-146b-3p inhibitor groups of colon cancer cells, while the knockdown of PRSS8 significantly reversed the effect of miR-146b-3p silencing on cell cycle (Figures 4(h) and 4(i), *P* < 0.05). Moreover, we also explored the effect of LINC00893 and miR-146b-3p on the expression of PRSS8. The results showed that LINC00893 overexpression significantly elevated PRSS8 expression, while miR-146b-3p overexpression significantly reduced the level of PRSS8 in colon cancer cells (Figures 4(j) and 4(k), *P* < 0.05).

### 3.5. Effects of LINC00893 in Colon Cancer *In Vivo*

**Figure 5:**
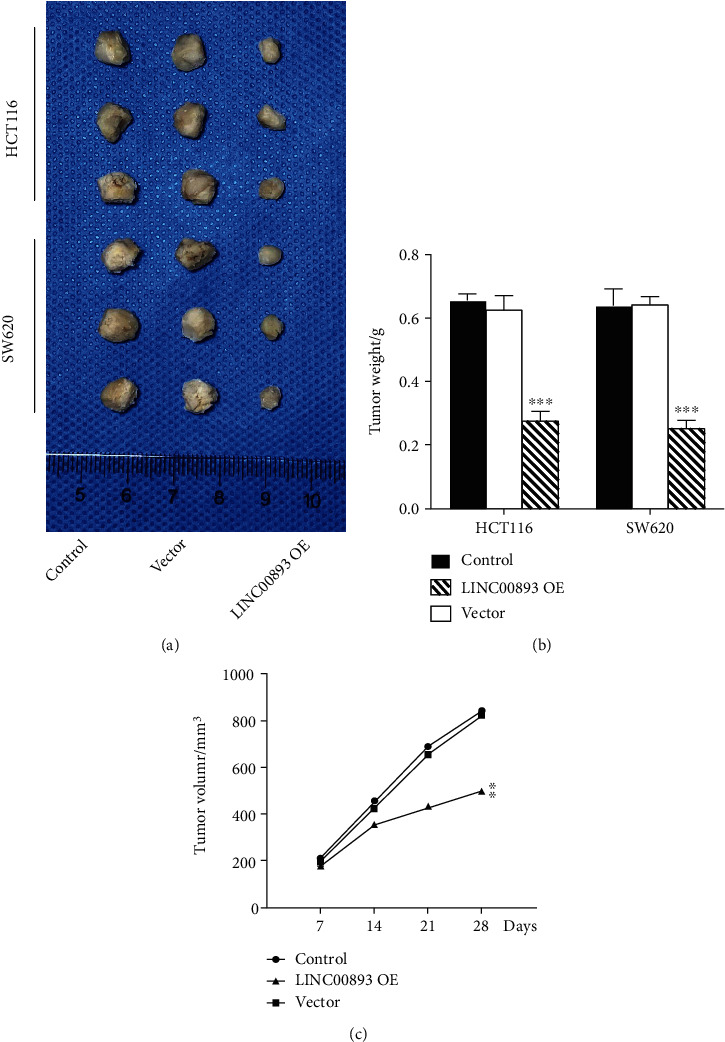
The evaluation of LINC00893 in colon cancer-based xenograft model utilizing HCT116 and SW620 cells. (a) The resected tumors of mice in three groups (LINC00893 OE, vector, and control). (b, c) Relative tumor weight and volume of mice in three groups were quantified. ^∗∗^*P* < 0.01, ^∗∗∗^*P* < 0.001.

To determine the effects of LINC00893 in colon cancer *in vivo*, the BALB/c colon cancer cell-based xenograft model was established using HCT116 and SW620 cells. Tumor weight and volume under the influence of LINC00893 OE were evaluated. Mice injected with LINC00893 OE showed significantly smaller tumor sizes *in vivo* compared with the vector and control group in Figure 5(a). The tumor weight and volume of mice were significantly reduced in LINC00893 OE group compared to other groups (Figures 5(b) and 5(c), *P* < 0.05).

## 4. Discussion

The disparity in the expression of genes across tumor tissues and normal tissues is important for reflecting cancer biology [29]. Previous investigations have shown that many lncRNAs are aberrantly expressed in tumors compared with that in normal tissues [29–31]. Basic characteristics of tumorigenesis include the ability to maintain proliferation, to activate invasion and metastasis, to induce angiogenesis, and to resist apoptosis [32]. In certain cases, lncRNAs may interfere with or inhibit cell growth [33] or engage in the complex interaction of malignant cells [34]. In addition, lncRNAs have been shown to exert impact on cell angiogenesis [34]. lncRNAs have the potential to be used as potential biomarkers for the diagnosis, prognosis, and therapy of a variety of diseases.

lncRNAs are known to be involved in several regulatory processes [35]. In colon cancer, many lncRNAs were irregularly expressed [36]. The lncRNA RP11 is discovered to be diminished in colorectal cancer tissues and is substantially connected to tumor metastases [4]. Furthermore, upregulation of MALAT1 enhances HIF-1 and endothelial cell protein levels, which influence colon cancer progression [37]. Our study had determined and confirmed the dysregulated expression of lncRNA LINC00893 in colon cancer tumors and adjacent tissues samples. Meanwhile, lncRNA regulates miRNA, which governs protein expression and cell function [38]. Researchers have found numerous human miRNAs, which modulate gene expression levels [39]. The link between miRNAs and colon cancer has gained attention. miRNAs would be potential colon cancer therapy targets [40]. The miR-146b-5p, miR-21, and miR-221 have been linked to lung cancer development and metastasis [30]. miR-146b-3p is a prognostic marker for various tumor types and has been found in several studies [41, 42]. Besides, PRSS8 is a tumor suppressor that has been shown to have essential roles in the prevention of colorectal cancer development and metastasis [43]. Present study has revealed the up-regulated expression of miR-146b-3p in colon cancer and its downregulation on PRSS8. LINC00893 is demonstrated to be downregulated in tumor tissues and cell lines of colon cancer. On the other hand, LINC00893 took part in colon cancer tumorigenesis by binding with miR-146b-3p to upregulate PRSS8. Moreover, our study found that lncRNA LINC00893, miR-146b-3p, and PRSS8 were valuable in distinguishing colon cancer tumors and adjacent tissue samples. Furthermore, the tumor weight and volume of bxenograft earing mice were significantly reduced after LINC00893 overexpression. Thus, LINC00893, miR-146b-3p, and PRSS8 genes might take part in colon cancer pathogenesis, providing novel diagnostic biomarkers of colon cancer.

Previous research showed that lncRNA LINC00662 overexpression targeting miR-340-5p/CLDN8/IL22 axis promoted proliferation, migration, and invasion and suppressed apoptosis of colon cancer cells via activating the ERK signaling pathway *in vitro* and *in vivo* [44]. Similarly, lncRNA HAGLR targeting the miR-185-5p/CDK4/CDK8 axis promoted proliferation, migration, and invasion of colon cancer *in vitro* and *in vivo* [45]. Our study has evaluated the biological functions and molecular regulations of LINC00893 in colon cancer cells. Overexpression of LINC00893 or miR-146b-3p inhibition suppressed cell growth, migration, and invasion of HCT116 and SW620 colon cancer cells and promoted the apoptotic activity of cells. Whereas miR-146b-3p overexpression reversed the antitumor effect of LINC00893 upregulation on colon cancer. PRSS8 knockdown rescued the inhibitory effect of miR-146b-3p knockdown on colon cancer progression.

Nonetheless, the current study has some limitations. First, the determination of LINC00893 was screened by an online database, which may present biased microarray results or samples. Second, detection of LINC00893, miR-146b-3p, and PRSS8 was conducted in a small sample size-based cohort, and therefore, future studies are needed for validation in a larger size-based cohort. Third, the present study did not reveal more clinical characteristics and risk factors of colon cancer patients. Fourth, the function and molecular mechanisms of LINC00893 were evaluated using only two cell lines without further validation of other up/downregulated genes involved in signaling pathways. Thus, further studies are needed to determine and assess LINC00893, miR-146b-3p, and PRSS8 in detail from various aspects.

## 5. Conclusion

LINC00893 overexpression can suppress the progression of colon cancer by binding with miR-146b-3p to upregulate PRSS8. LINC00893, miR-146b-3p, and PRSS8 may serve as novel biomarkers and therapeutic targets of colon cancer, providing new treatment options and research approaches towards colon cancer.

## Figures and Tables

**Table 1:** Correlation of the LINC00893 expression with clinical characteristics in the colon cancer patients (*n* = 30).

Variables	LINC00893 expression		*P*
	Low	High	
Age			0.6377
< 60 years	9	8	
≥ 60 years	8	5	
Gender			0.7851
Male	7	6	
Female	10	7	
Location			0.6426
Left	12	6	
Right	7	5	
Histology			0.4906
Adenocarcinoma	10	6	
Mucinous adenocarcinoma	7	7	
Stage			0.0001
I + II	8	2	
III + IV	2	18	
T stage			0.0704
Tis	11	5	
T1-T3	5	9	
N stage			0.4642
No	7	8	
N1 + N2	9	6	
M stage			0.1266
M0	11	2	
M1	10	7	
Status			0.8655
Death	3	2	
Alive	16	9	

## Data Availability

Data archiving will be made available on reasonable request, all of the authors are responsible to the data.
